# Severe Hyperosmotic Stress Issues an ER Stress-Mediated “Death Sentence” in H9c2 Cells, with p38-MAPK and Autophagy “Coming to the Rescue”

**DOI:** 10.3390/biomedicines10061421

**Published:** 2022-06-15

**Authors:** Konstantina-Eleni Bourouti, Christos Konstantaros, Catherine Gaitanaki, Ioanna-Katerina Aggeli

**Affiliations:** Section of Animal and Human Physiology, Faculty of Biology, School of Science, National and Kapodistrian University of Athens, University Campus, Ilissia, 157 84 Athens, Greece; kebourouti@gmail.com (K.-E.B.); ckonstantaros@gmail.com (C.K.); cgaitan@biol.uoa.gr (C.G.)

**Keywords:** hyperosmotic stress, H9c2 cells, IRS, ER stress, autophagy, oxidative stress, eIF2α, apoptosis

## Abstract

With several cardiovascular pathologies associated with osmotic perturbations, researchers are in pursuit of identifying the signaling sensors, mediators and effectors involved, aiming at formulating novel diagnostic and therapeutic strategies. In the present study, H9c2 cells were treated with 0.5 M sorbitol to elicit hyperosmotic stress. Immunoblotting as well as cell viability analyses revealed the simultaneous but independent triggering of multiple signaling pathways. In particular, our findings demonstrated the phosphorylation of eukaryotic translation initiation factor 2 (eIF2α) and upregulation of the immunoglobulin heavy-chain-binding protein (BiP) expression, indicating the onset of the Integrated Stress Response (IRS) and endoplasmic reticulum stress (ERS), respectively. In addition, autophagy was also induced, evidenced by the enhancement of Beclin-1 protein expression and of AMP-dependent kinase (AMPK) and Raptor phosphorylation levels. The involvement of a Na^+^/H^+^ exchanger-1 (NHE-1) as well as NADPH oxidase (Nox) in 0.5 M sorbitol-induced eIF2α phosphorylation was also indicated. Of note, while inhibition of ERS partially alleviated the detrimental effect of 0.5 M sorbitol on H9c2 cellular viability, attenuation of p38-MAPK activity and late phase autophagy further mitigated it. Deciphering the mode of these pathways’ potential interactions and of their complications may contribute to the quest for effective clinical interventions against associated cardiovascular diseases.

## 1. Introduction

Cells, tissues and organs maintain a strict, multilevel regulatory mechanism of their osmotic equilibrium, since preservation of the latter is tightly associated with their proper function and viability. Nevertheless, οsmotic perturbations may occur locally or in a systemic manner, triggering adaptive responses to reinstate cellular volume [1]. Failure of these mechanisms to counterbalance severe osmotic variations has been established to cause a number of pathologies, including cardiovascular disorders [2], i.e., myocardial infarction, hypoxia, ischemia/reperfusion injury and vasculopathies [3]. With cardiovascular diseases being the leading cause of death worldwide, probing into the cellular responses triggered by osmotic perturbations in cardiac cells is of great significance.

In eukaryotic cells, various stressors have been demonstrated to activate the Integrated Stress Response (IRS), an intricate signaling pathway aiming at restoring homeostasis [4]. In this context, protein synthesis is suppressed, with a limited number of genes induced, in an effort to promote cell recovery. Depending on the duration, nature and intensity of the stress stimulus, activation of the IRS may either exert a beneficial effect, re-establishing homeostasis, or may elicit a detrimental outcome, favoring cell death [5]. Accordingly, accumulating data have demonstrated a link between IRS and pro-survival pathways, including: the Unfolded Protein Response (UPR) and autophagy, but also an interplay with cell death mechanisms, mainly apoptosis and necrosis [4]. Of note, IRS can be induced either by exogenous or endogenous stress stimuli, i.e., by endoplasmic reticulum stress (ERS). Interestingly, ERS has been recently shown to mediate hyperosmotic-stress-induced apoptosis in cardiac cells [6].

Upon stimulation of ERS, a number of cellular functions are compromised, with impairment of protein structural maturation resulting in the accumulation of misfolded proteins, ultimately leading to activation of the UPR [7]. The three major UPR activators (IRE1, PERK and ATF6) subsequently initiate separate branches, promoting the removal of improperly folded proteins [8]. The signaling pathways triggered, contributing to the restoration of homeostasis, involve: (a) increased transcription of genes encoding for ER chaperones, such as the immunoglobulin heavy-chain-binding protein (BiP) that increases the folding capacity of the ER or (b) phosphorylation of the eukaryotic translation initiation factor eIF2α [9]. Although the UPR is an adaptive response established to alleviate ERS, if activated over a prolonged time, it has been found to promote apoptosis [10].

Osmotic stress has also been established to disturb cellular proteostasis [11] due to the accumulation of misfolded or oxidized proteins, an effect relieved by protein turnover mechanisms, including autophagy [12]. During autophagy, double membrane structures, called autophagosomes, engulf cytoplasmic components, such as aggregated or damaged proteins and organelles. Subsequently, autophagosomes fuse with lysosomes and their cargo is degraded [12]. Particularly in the heart, studies have shown that autophagy mediates myocardial adaptation to oxidative stress via removal of malfunctioning proteins or mitochondria. Upon activation, AMP-dependent kinase (AMPK), a major energy sensor protein, fine tunes the autophagic mechanism [13]. Nevertheless, even though autophagy elicits a cardioprotective effect, it may also induce cell death under extreme conditions [14].

Hence, in the present study, we dissected the signaling mechanisms triggered by hyperosmotic stress in H9c2 cells. The latter represent an established experimental setting used in mechanistic studies that aim to unravel the key effectors of cellular responses under conditions of stress in cardiac cells. To this end, H9c2 cells were treated with 0.5 M sorbitol, a routinely used stimulant of hyperosmotic stress in cardiac cells [15]. Sorbitol is generated via conversion of glucose by aldose reductase, in the initial step of the polyol pathway, an alternative route to glucose metabolism. It is next converted to fructose by sorbitol dehydrogenase [16]. Accumulation of sorbitol causes osmotic stress, simulating the detrimental complications of diabetes on myocardial contractility and function. Under such severe osmotic fluctuations, a network of signaling pathways is activated that either independently or synergistically dictates to the cells whether to live or die. Since the potential interplay of the responses triggered under these conditions remains unresolved, delineating the role of the molecular mechanisms enacted appears of exquisite significance. Data derived here could contribute to the quest for novel biomarkers or drug targets and development of groundbreaking treatments against cardiovascular disorders.

## 2. Materials and Methods

### 2.1. Reagent and Antibodies

Dithiothreitol (DTT), phenylmethylsulphonyl fluoride (PMSF), dimethyl sulfoxide (DMSO), D-sorbitol and Bradford protein assay reagent were purchased from AppliChem GmbH (Darmstadt, Germany). Leupeptin, trans-epoxysuccinyl-L-leucylamido-(4-guanidino) butane (E-64), trypan blue (0.4% *w*/*v*) and SB203580 were purchased from Merck (Burlington, MA, USA). HOE642, apocynin, bafilomycin A1 and 4-Phenylbutyric acid (4-PBA) were purchased from Sigma-Aldrich (St. Louis, MO, USA).

Nitrocellulose (0.45 μm) was obtained from Macherey-Nagel GmbH (Duren, Germany). Prestained molecular mass markers were from New England Biolabs (Beverly, MA, USA). The rabbit polyclonal antibodies for: poly (ADP-ribose) polymerase (PARP) (#9542), caspase-3 (#9662), Beclin-1 (#3495), BiP (#3183), eIF2α (#9722), LC3B (#2775), phospho-AMPKα (Thr172) (#2535), AMPKα (#5831), phospho-eIF2α (Ser51) (#9721), phospho-p38-MAPK (Thr180/Tyr182) (#9211), p38-MAPK (#9212), phospho-p44/42 MAPK (Erk1/2) (Thr202/Tyr204) (#9101), p44/42 MAPK (Erk1/2) (#9102), phospho-JNK1/2 (Thr183/Tyr185) (#9251), JNK1/2 Antibody (#9252) and phospho-Raptor (Ser792) (#2083) were purchased from Cell Signaling Technology Inc. (Beverly, MA, USA), while rabbit polyclonal anti-actin (A2103) was from Merck (Burlington, MA, USA). SB203580, SP600125 and PD98059 were also from Merck. The peroxidase-conjugated goat anti-rabbit IgG secondary antibody (#AP132P) was from Merck. The enhanced chemiluminescence (ECL) kit was from GE Healthcare (Buckinhamshire, UK). Super RX film was purchased from Fuji photo film GmbH (Dusseldorf, Germany). Cell culture supplies were from PAA Laboratories (Pasching, Austria). Oxyblot Protein Oxidation Detection Kit was from Merck and 5-(and-6)-chloromethyl-2′,7′-dichloro-dihydro-fluorescein diacetate, acetyl ester (CM-H_2_DCFDA) was purchased from Invitrogen, Thermo Fisher Scientific (Waltham, MA, USA).

### 2.2. Cell Culture and Treatments

H9c2 rat cardiac myoblasts (passage 18–25; American Type Culture Collection CRL-1446, Manassas, VA, USA) were grown in medium containing high glucose (4.5 g/L) Dulbecco’s modified Eagle’s medium (DMEM; Gibco, Paisley, UK) in the presence of 10% (*v*/*v*) fetal bovine serum (FBS; ThermoFischer Scientific, Waltham, MA, USA) and penicillin-streptomycin (ThermoFischer Scientific, Waltham, MA, USA), under a humidified atmosphere of 95% air/5% CO_2_ at 37 °C. Cells were seeded in 60 mm dishes and grown to approximately 70% confluence. Before performing any treatment, serum was withdrawn for at least 18 h. Cells were treated with sorbitol (0.5 M) for the times indicated. Apart from 4-PBA which was dissolved in methanol, the pharmacological inhibitors used were dissolved in DMSO and added to the medium 30 min prior to treatment with sorbitol. Thus, H9c2 cells were left untreated (control), incubated with the inhibitors alone, or with the inhibitors followed by exposure to sorbitol, for the times indicated.

### 2.3. Cell Viability Assays

The number of viable cells was determined using the Trypan Blue exclusion assay. Trypan Blue Staining Solution is a vital stain that traverses the membrane of a non-viable cell, coloring it blue. Hence, after seeding H9c2 cells in 60 mm dishes, cells were left untreated (control), treated with the inhibitor compounds alone or with the inhibitors followed by exposure to sorbitol (0.5 M). All experiments were performed in triplicate. After treatment, cells were rinsed with PBS and harvested with trypsin. To determine the percentage of viable cells, the cell suspension was subsequently mixed with 0.4% (*w*/*v*) trypan blue solution (in a 5:1 ratio) and incubated for 2 min, before loading onto a hemacytometer and counted under a light microscope. Percentage of viable cells was calculated as follows:

% viable cells = (number of unstained cells/total number of cells) × 100

The number of viable cells was also determined using the 3-(4,5-dimethylthiazol-2-yl)-2,5-diphenyltetrazolium bromide (MTT) assay. After seeding cells in 96-well culture plates (5 × 10^3^ cells/well), they were left untreated (control), treated with the inhibitor compounds alone or with the inhibitors followed by exposure to sorbitol (0.5 M). All experiments were performed in triplicate. After incubation with 50 μg MTT per well, the medium was aspirated, cells were lysed in 0.1 M HCl/isopropanol to dissolve the reduced MTT formazan crystals and absorbance was measured in an ELISA microplate reader (DENLEY, West Sussex, UK) using a 545 nm filter. All experiments were performed in triplicate.

### 2.4. Protein Extraction

After treatments, cells were washed twice with ice-cold phosphate-buffered saline (PBS) and harvested. For whole cell extracts, H9c2 cells were lysed in ice-cold buffer (20 mM Tris-HCl pH 7.5, 20 mM β-glycerophosphate, 2 mM EDTA, 10 mM benzamidine, 20 mM NaF, 0.2 mM Na_3_VO_4_, 200 μM leupeptin, 10 μM E-64, 5 mM DTT, 300 μM PMSF and 0.5% (*v*/*v*) Triton X-100) and subsequently incubated on ice for 15 min. After lysates were centrifuged (BR4i Jouan centrifuge, 20,800× *g*, 10 min, 4 °C), the supernatants (total protein extract) were collected. Protein concentration was determined using the Bradford assay. After quantification, 0.33 vol. of sodium dodecyl sulphate (SDS) sample buffer (SB4X: 0.33 mol/L Tris-HCl (pH 6.8), 10% (*w*/*v*) SDS, 13% (*v*/*v*) glycerol, 20% (*v*/*v*) 2-mercaptoethanol and 0.2% (*w*/*v*) bromophenol blue) was added to the samples which were next boiled and stored until use at −20 °C. When preparing the samples for detection of caspase-3 activation, Chaps buffer (50 mM HEPES/KOH pH 6.5, 2 mM EDTA, 0.1% (*w*/*v*) Chaps, 20 μg/mL leupeptin, 5 mM DDT, 1 mM PMSF, 10 μg/mL aprotinin and 10 μg/mL pepstatin A) was used. After homogenization, samples were repeatedly frozen (−80 °C (×3)) and left to thaw. Once more, after centrifugation of lysates (20,800× *g*, 4 °C, 20 min), protein concentrations were assessed using the Bradford assay. Samples were next boiled with 0.33 vol. of SB4X.

### 2.5. Oxyblot Protein Oxidation Detection

Protein oxidation was detected by reaction with 2,4-dinitrophenyl hydrazine (DNP) using an OxyBlot™ Protein Oxidation Detection Kit (Merck). Briefly, after seeding H9c2 cells in 60 mm dishes, cells were left untreated (control), treated with apocynin alone, or with apocynin followed by exposure to sorbitol (0.5 M), or treated with sorbitol (0.5 M) for 1 h or with diamide (2 mM) for 8 h. All experiments were performed in triplicate. After treatments, cells were rinsed with PBS, homogenized in RIPA buffer (50 mM Tris-HCl pH 7.5, 150 mM NaCl, 1% (*v*/*v*) Triton X-100 and 0.1% SDS) and incubated on ice for 15 min. Lysates were centrifuged in a BR4i Jouan centrifuge (14,000× *g*, 5 min, 4 °C) and the supernatants were collected. Protein concentration was determined using the Bradford assay. Carbonyl groups in the protein side chains were derivatized to DNP-hydrazone by reaction with DNPH. Proteins were next electrophoresed on an SDS-PAGE gel, followed by immunoblotting with an anti-DNP antibody (1:150) so as to detect carbonyl groups.

### 2.6. Intracellular ROS Detection

Intracellular generation of ROS was assessed by the redox-sensitive and membrane-permeable fluorescent probe 5-(and-6)-chloromethyl-2′,7′-dichloro-dihydro-fluorescein diacetate, acetyl ester (CM-H_2_DCFDA). In brief, H9c2 cells seeded in 60 mm dishes were left untreated (control) or were treated with sorbitol (0.5 M) for 1 h, or with tBHP (50 μM) for 1 h. Subsequently, cells were incubated with CM-H_2_DCFDA (10 μM) at 37 °C for 30 min in the dark. They were then washed twice with PBS and lysed with cold lysis buffer as described in the preparation of protein extracts. The homogenates were next centrifuged at 10,000× *g* for 5 min to remove cell debris. Fluorescence intensity was measured using a fluorescence reader (VersaFluorTM, BIO-RAD Hercules, CA, USA) with an excitation filter of 490 nm and an emission filter of 520 nm. Treatments were done in replicates of three and three independent experiments were performed.

### 2.7. SDS-PAGE and Immunoblot Analysis

Protein samples containing equal amounts of protein (40 μg) were resolved by SDS-PAGE on 8% (*w*/*v*), 10% (*w*/*v*) or 12% (*w*/*v*) polyacrylamide gels and transferred onto nitrocellulose membranes (0.45 μm). After blocking in TBST (Tris-buffered saline Tween) containing 5% (*w*/*v*) non-fat milk powder (60 min, room temperature), membranes were incubated overnight with the appropriate antibody, according to the manufacturer’s instructions (at 1:1000 dilution). After incubation with the respective horseradish peroxidase-conjugated secondary antibody (1:5000 dilution), blots were developed using enhanced chemiluminescence (ECL) and quantified by scanning densitometry (Gel Analyzer v. 1.0). Equal protein loading was verified by probing membranes with an anti-actin antibody. Normalization was carried out by dividing the average value of each protein studied, with the respective levels of the control protein in each sample.

### 2.8. Statistical Evaluations

Western blots shown are representative of at least three independent experiments. Data shown correspond to the mean ± SEM and were analyzed by one-way ANOVA multiple comparison test (Graph Pad Prism Software, San Diego, CA, USA) with group comparisons performed using the Bonferroni post-hoc test. *p* < 0.05 was considered to indicate a statistically significant difference.

## 3. Results

### 3.1. Hyperosmotic Stress Induces eIF2α Phosphorylation and BiP Expression Levels in H9c2 Cells

**Figure 1:**
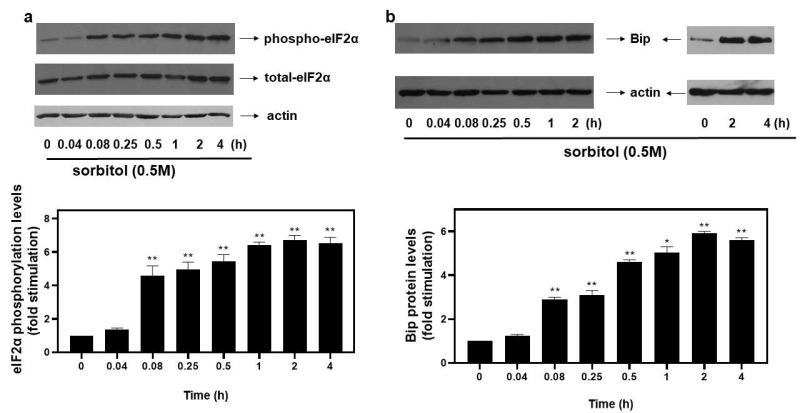
Kinetics of hyperosmotic-stress–induced phosphorylation of eIF2α in H9c2 cardiac cells. H9c2 cells were exposed to 0.5 M sorbitol for the times indicated. Protein extracts (40 μg/lane) were subjected to SDS-PAGE and immunoblotted with antibodies for phosphorylated eIF2α ((**a**), upper panel), total levels of eIF2α ((**a**) middle panel), total levels of BiP ((**b**) upper panel) and actin ((**a**,**b**) bottom panels). Western blots presented are representative of at least three independent experiments with overlapping results. Immunoreactive bands were quantified by scanning densitometry and plotted ((**a**,**b**) respective graphs). Results are means ± SEM for at least three independent experiments. * *p* < 0.05, ** *p* < 0.01 compared to control values.

Upon activation of the ISR pathway under stress conditions, the alpha subunit of eukaryotic translation initiation factor 2 (eIF2α) is phosphorylated at Ser51, rendering it inactive and, hence, suppressing initiation of translation [4]. In our experimental setting, exposure of H9c2 cells to 0.5 M sorbitol promoted phosphorylation of eIF2α at as early as 5 min (4.59 ± 0.58-fold relative to control, *p* < 0.01). Phosphorylation levels of eIF2α were maximized at 2 h (6.74 ± 0.27-fold relative to control, *p* < 0.01), remaining elevated for at least 4 h (6.53 ± 0.35-fold relative to control, *p* < 0.01) (Figure 1a, top panel and graph). No significant change was detected in the levels of total eIF2α during these interventions (Figure 1a, middle panel). Equal protein loading was verified by immunoblotting analysis of actin levels (Figure 1a, bottom panel).

With osmotic stress shown to induce ER stress in cardiac cells [6], we next assessed protein levels of BiP, a major ER chaperone. Thus, treatment of H9c2 cells with 0.5 M sorbitol caused an immediate increase in BiP expression levels observed from 5 min (2.9 ± 0.1-fold relative to control, *p* < 0.01), maximizing at 2 h (5.91 ± 0.09-fold relative to control, *p* < 0.01) and remaining significantly elevated for 4 h (5.61 ± 0.11-fold relative to control, *p* < 0.01) (Figure 1b, top panels and graph). Equal protein loading was verified by immunoblotting analysis of actin levels (Figure 1b, bottom panels).

### 3.2. Hyperosmotic Stress Promotes Autophagy in H9c2 Cells

**Figure 2:**
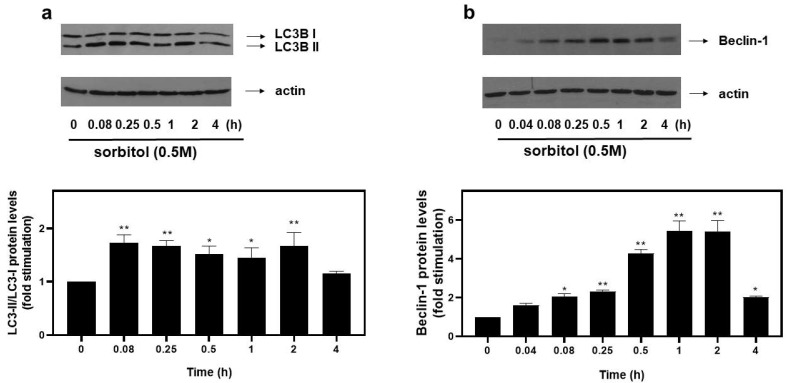
Hyperosmotic stress induces an increase in autophagic markers in H9c2 cardiac cells. H9c2 cells were exposed to 0.5 M sorbitol for the times indicated. Protein extracts (40 μg/lane) were subjected to SDS-PAGE and immunoblotted with antibodies for endogenous levels of total LC3B protein (LC3 I and LC3 II forms) ((**a**) upper panel), actin ((**a**,**b**) bottom panels) and total levels of Beclin-1 ((**b**) upper panel). Western blots presented are representative of at least three independent experiments with overlapping results. Immunoreactive bands were quantified by scanning densitometry and plotted ((**a**,**b**) respective graphs). Results are means ± SEM for at least three independent experiments. * *p* < 0.05 and ** *p* < 0.01 compared to control values.

Emerging studies reveal upregulation of the autophagic mechanism under hyperosmotic conditions [12]. During autophagy, LC3 (microtubule-associated protein light chain 3) is lipidated and converted from its cytoplasmic LC3-I to its LC3-II form, with their ratio routinely used as an indicator of autophagosome formation [17]. Therefore, in order to monitor the autophagic flux potentially initiated in H9c2 cells exposed to 0.5 M sorbitol, we subsequently looked into the LC3-II/LC3-I ratio. As shown in Figure 2a (top panel and respective graph), maximal values of the LC3-II/LC3-I ratio were attained at 5 min (1.73 ± 0.15-fold relative to control, *p* < 0.01), remaining elevated for at least 2 h (1.67 ± 0.26-fold relative to control, *p* < 0.01). Equal protein loading was confirmed by immunodetection of actin levels (Figure 2a, bottom panel).

To further substantiate the occurrence of autophagy, protein levels of Beclin-1, a major autophagy effector [18], were examined. Hence, we observed a rapid increase in the expression levels of Beclin-1 at 5 min (2.01 ± 0.22-fold relative to control, *p* < 0.05), with maximal levels at 1–2 h (5.43 ± 0.53-fold relative to control, *p* < 0.01), declining thereafter (2.03 ± 0.05-fold relative to control) (Figure 2b, top panel and graph). Once again, equal protein loading was verified by immunoblotting analysis of actin levels (Figure 2b, bottom panel).

**Figure 3:**
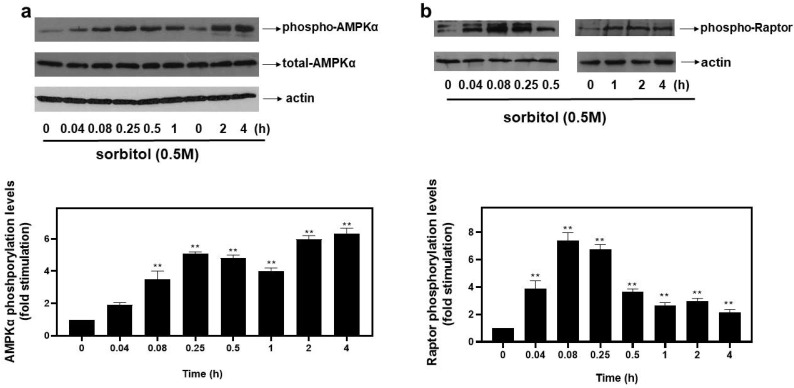
Hyperosmotic stress induces phosphorylation of the alpha catalytic subunit of AMPK (AMPKα) and Raptor in H9c2 cardiac cells. H9c2 cells were exposed to 0.5 M sorbitol for the times indicated. Protein extracts (40 μg/lane) were subjected to SDS-PAGE and immunoblotted with antibodies for phosphorylated AMPKα (**a** upper panel), total levels of AMPKα ((**a**) middle panel), phosphorylated Raptor ((**b**) upper panel) and actin ((**a**,**b**) bottom panels). Western blots presented are representative of at least three independent experiments with overlapping results. Immunoreactive bands were quantified by scanning densitometry and plotted ((**a**,**b**) respective graphs). Results are means ± SEM for at least three independent experiments. ** *p* < 0.01 compared to control values.

Accumulating evidence on the mechanisms that induce autophagy under stress conditions highlights the role of AMP-dependent kinase (AMPK) [1] and the mechanistic target of rapamycin (mTOR) [19]. Raptor, an established substrate of AMPK, is a subunit of mTOR complex 1 (mTORC1) and its phosphorylation at serine 792 by AMPK inactivates mTORC1, resulting in further enhancement of autophagy [13]. Thus, the potential involvement of AMPK and Raptor in the observed responses was assessed by probing into their phosphorylation profiles. As illustrated in Figure 3a (upper panel and graph), hyperosmotic stress induced a rapid and pronounced phosphorylation of threonine 172 in the alpha catalytic subunit of AMPK (AMPKα), which renders the kinase active [1]. The phosphorylation was evident 5 min after the onset of the treatment (3.47 ± 0.33-fold relative to control, *p* < 0.01) and was maximized at 4 h (6.33 ± 0.33-fold relative to control, *p* < 0.01). Immunoblot analysis of AMPKα total levels revealed its constitutive expression (Figure 3a, middle panel). Equal protein loading was once more verified by detecting actin levels (Figure 3a, bottom panel). As far as Raptor is concerned, its phosphorylation was maximized at 5 min (7.4 ± 0.4-fold relative to control, *p* < 0.01), gradually declining thereafter (Figure 3b, top panels and graph). Equal protein loading was verified by probing with the specific anti-actin antibody (Figure 3b, bottom panels).

### 3.3. Hyperosmotic-Stress-Induced eIF2α Phosphorylation Is p38-MAPK Independent and ROS Mediated

**Figure 4:**
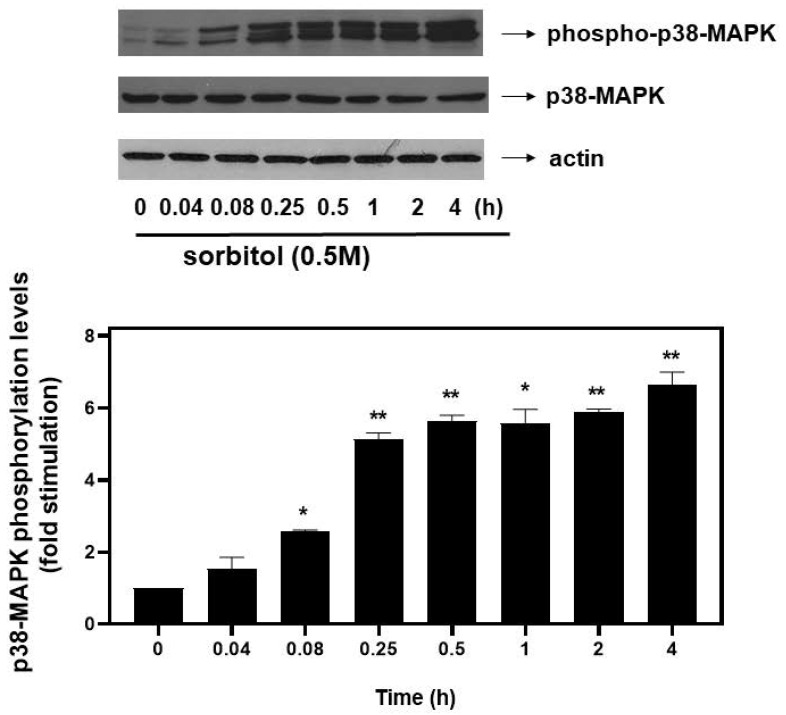
Time-dependent profile of hyperosmotic-stress-induced p38-MAPK phosphorylation in H9c2 cardiac cells. H9c2 cells were exposed to 0.5 M sorbitol for the times indicated. Protein extracts (40 μg/lane) were subjected to SDS-PAGE and immunoblotted with antibodies for phosphorylated p38-MAPK (**upper** panel), for total levels of p38-MAPK (**middle** panel) and for total actin levels (**bottom** panel). Western blots presented are representative of at least three independent experiments with overlapping results. Immunoreactive bands were quantified by scanning densitometry and plotted (respective graph). Results are means ± SEM for at least three independent experiments. * *p* < 0.05 and ** *p* < 0.01 compared to control values.

An effort was next made to identify possible signaling effectors involved in the hyperosmotic-stress-induced phosphorylation of eIF2α. Given that members of the mitogen-activated protein kinases (MAPKs) family have been shown to function as principal signal transduction mediators in cardiac cells under osmotic stress conditions [20], we set out to investigate their potential involvement in eIF2α phosphorylation. First, in accordance with previous reports demonstrating all three best-studied MAPK family members’ phosphorylation, thus, activation by osmotic stress [21], we also found that treatment of H9c2 cells with 0.5 M sorbitol for 5 min up to 4 h resulted in a time-dependent activation of all three MAPK members. In particular, we observed a significant increase in p38-MAPK phosphorylation levels from as early as 5 min (2.57 ± 0.04-fold relative to control, *p* < 0.05) after exposure of H9c2 cells to 0.5 M sorbitol, gradually attaining maximal values after 4 h (6.65 ± 0.35-fold relative to control, *p* < 0.01) (Figure 4, upper panel and graph). The dual phosphorylation of p38-MAPK at threonine 180 and tyrosine 182, detected by this specific antibody, is essential for its activation [21]. Thus, the triplet detected could correspond to differentially phosphorylated forms of p38-MAPK migrating at different positions, or to different p38-MAPK isoforms. Further studies are required to clarify and decipher the nature of the bands detected. Immunoblot analysis using an antibody against total p38-MAPK levels revealed no fluctuations (Figure 4, middle panel). Equal protein loading was verified by probing for actin levels (Figure 4, bottom panel).

**Figure 5:**
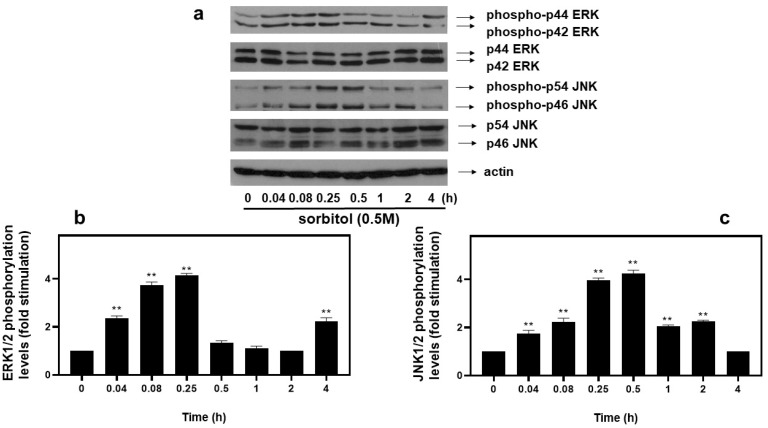
Time-dependent profile of hyperosmotic-stress-induced ERK1/2 and JNK1/2 phosphorylation in H9c2 cardiac cells. H9c2 cells were exposed to 0.5 M sorbitol for the times indicated. Protein extracts (40 μg/lane) were subjected to SDS-PAGE and immunoblotted with antibodies for phosphorylated ERK1/2 ((**a**) upper panel), for total levels of ERK1/2 ((**a**) 2nd panel from top), for phosphorylated JNK1/2 ((**a**) 3rd panel from top), for total levels of JNK1/2 ((**a**) 4th panel from top) and for total actin levels ((**a**) bottom panel). Western blots presented are representative of at least three independent experiments with overlapping results. Immunoreactive bands were quantified by scanning densitometry and plotted ((**b**,**c**) respective graphs). Results are means ± SEM for at least three independent experiments. ** *p* < 0.01 compared to control values.

As far as extracellular-signal-regulated kinases (ERKs) and c-Jun NH_2_-terminal kinases (JNKs) are concerned, we detected an equally rapid upregulation of their phosphorylation levels from as early as 5 min after exposure of H9c2 cells to 0.5 M sorbitol, with maximal values attained after 15 min for ERKs (4.13 ± 0.15-fold relative to control, *p* < 0.01) (Figure 5a, upper panel and b) and after 30 min for JNKs (4.25 ± 0.25-fold relative to control, *p* < 0.01) (Figure 5a, third panel from top and c). No significant fluctuations were observed in total levels of ERKs or JNKs under the conditions investigated (Figure 5a: 2nd and 4th panels from top, respectively). Equal protein loading was verified by immunoblotting identical samples with a specific anti-actin antibody (Figure 5a, bottom panel).

**Figure 6:**
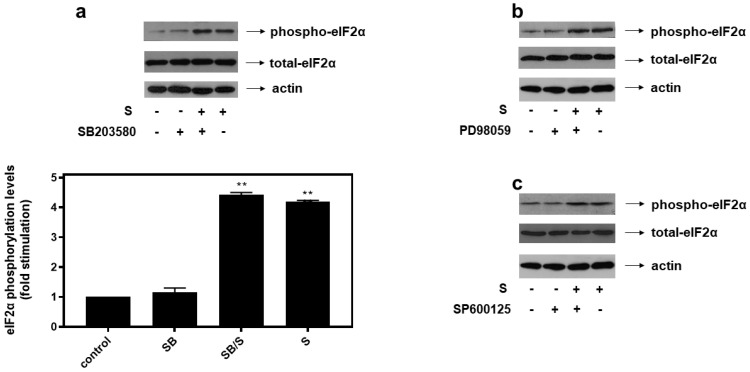
p38-MAPK (**a**), ERK1/2 (**b**) and JNK1/2 (**c**) are not involved in hyperosmotic-stress-induced phosphorylation of eIF2α in H9c2 cardiac cells. H9c2 cells were left untreated (control), or treated with 0.5 M sorbitol (S), or incubated with SB203580 (SB), PD98059, SP600125, or were pre-incubated with SB203580, PD98059, SP600125 for 30 min and then exposed to 0.5 M sorbitol in the presence of SB203580 (SB/S), PD98059 and SP600125, respectively. Cell extracts (40 μg/lane) were subjected to SDS-PAGE and immunoblotted with an antibody that detects phosphorylated eIF2α (upper panels), total levels of eIF2α (middle panels) as well as total levels of actin (bottom panels). Western blots are representative of at least three independent experiments with overlapping results. Immunoreactive bands were quantified by laser scanning densitometry and plotted (respective graph). Results are means ± SEM for at least three independent experiments. ** *p* < 0.01 compared to control values.

With all MAPK subfamilies activated by hyperosmotic stress in our experimental setting, H9c2 cells were subsequently left untreated (control-C) or were incubated with SB203580 (SB—10 μM), a p38-MAPK inhibitor, PD98059 (PD—25 μM), an ERK1/2 inhibitor, SP600125 (SP—10 μM), a JNK1/2 inhibitor, alone or with the inhibitors followed by exposure to 0.5 M sorbitol. As shown in Figure 6, pre-treatment of H9c2 cells with SB203580 (a), PD98059 (b) and SP600125 (c) had no effect on hyperosmotic-stress-induced eIF2α phosphorylation levels.

**Figure 7:**
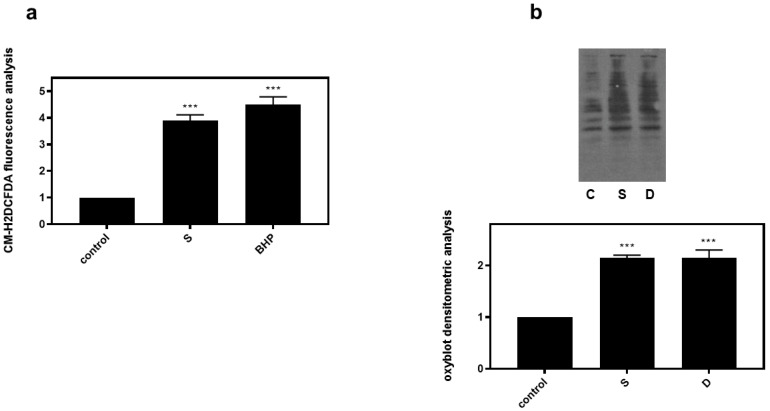
Hyperosmotic stress induces generation of ROS in H9c2 cardiac cells. H9c2 cells were exposed to 0.5 M sorbitol (S) or tBHP and were subsequently incubated with CM-H_2_DCFDA. After protein extraction the fluorescence intensity was measured in the homogenates (**a**). For oxyblot analysis H9c2 cells were exposed to 0.5 M sorbitol (S) or Diamide (D) and homogenized in RIPA buffer. Lysates were incubated with DNPH and proteins were electrophoresed (SDS-PAGE) and immunoblotted with an anti-DNP antibody (**b**). Immunoreactive bands were quantified by scanning densitometry and plotted ((**b**) respective graph). Results are means ± SEM for at least three independent experiments. *** *p* < 0.001 compared to control values.

Correlating with the established association between hyperosmotic perturbations and oxidative stress [22,23], 0.5 M sorbitol (S) was subsequently found to significantly increase dichlorofluorescein diacetate (DCFH-DA) fluorescence in H9c2 cells (3.9 ± 0.21-fold relative to control, *p* < 0.001), with 50 μM tBHP (BHP) (as positive control) exerting a similar effect (4.5 ± 0.29-fold relative to control, *p* < 0.001) (Figure 7a). Performing oxyblot analysis, we also observed that after treatment of H9c2 cells with 0.5 M sorbitol for 1 h, levels of carbonyl groups were considerably enhanced (2.15 ± 0.05-fold relative to control, *p* < 0.001), indicative of the oxidative modifications conferred by hyperosmotic stress (Figure 7b). Diamide (2 mM), used as a positive control, had a similar effect (2.01 ± 0.14-fold relative to control, *p* < 0.001) (Figure 7b).

**Figure 8:**
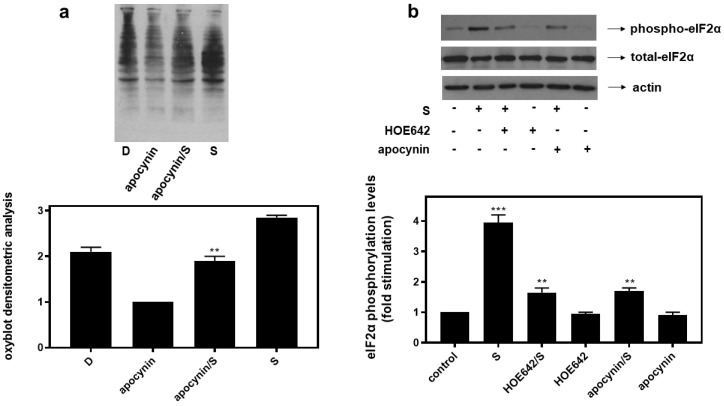
HOE642, a specific inhibitor of NH-1, as well as apocynin, an inhibitor of Nox, suppress hyperosmotic-stress-induced phosphorylation of eIF2α in H9c2 cardiac cells. (**a**) Oxyblot analysis was performed in H9c2 cells exposed to Diamide (D), apocynin, apocynin followed by sorbitol in the presence of apocynin (apocynin/S) and 0.5 M sorbitol (S). After homogenization in RIPA buffer, lysates were incubated with DNPH and proteins were electrophoresed (SDS-PAGE) and immunoblotted with an anti-DNP antibody. Immunoreactive bands were quantified by scanning densitometry and plotted ((**a**) respective graph). (**b**) H9c2 cells were left untreated (control) or were incubated with the inhibitors alone or with the inhibitors followed by exposure to 0.5 M sorbitol (S) in the presence of the inhibitors. Cell extracts (40 μg/lane) were subjected to SDS-PAGE and immunoblotted with an antibody that detects phosphorylated eIF2α (upper panel), total levels of eIF2α (middle panel) as well as total levels of actin (bottom panel). Western blots are representative of at least three independent experiments with overlapping results. Immunoreactive bands were quantified by laser scanning densitometry and plotted (respective graph). Results are means ± SEM for at least three independent experiments. *** *p* < 0.001 compared to control values; ** *p* < 0.01 compared to sorbitol-treated cells in the absence of the inhibitors.

Interestingly, inhibition of key enzymes in oxidative stress generation mitigated eIF2α phosphorylation in H9c2 cells treated with 0.5 M sorbitol (S) for 1 h. H9c2 cells were left untreated (control) or were incubated with the inhibitors alone or with the inhibitors followed by exposure to 0.5 M sorbitol for 1 h. Thus, HOE642 (5 μM), a specific inhibitor of Na^+^/H^+^ exchanger-1 (NHE-1), as well as apocynin (10 μM), known to inhibit NADPH oxidase (Nox) and shown to partially reverse sorbitol-induced levels of carbonyl groups in the oxyblot analysis (Figure 8a), both markedly reduced phosphorylation levels of eIF2α by approximately 58.23 ± 0.29% and 56.96 ± 0.18%, respectively (Figure 8b, upper panel and graph, *p* < 0.01). Immunodetection of total eIF2α protein levels revealed, once again, their constitutive expression (Figure 8b, middle panel). Equal protein loading was verified by immunoblotting analysis of actin levels (Figure 8b, bottom panel).

### 3.4. p38-MAPK and Autophagy “Come to the Rescue” of H9c2 Cells Exposed to Hyperosmotic Stress, with ER Stress Contributing to the Induced Apoptosis

**Figure 9:**
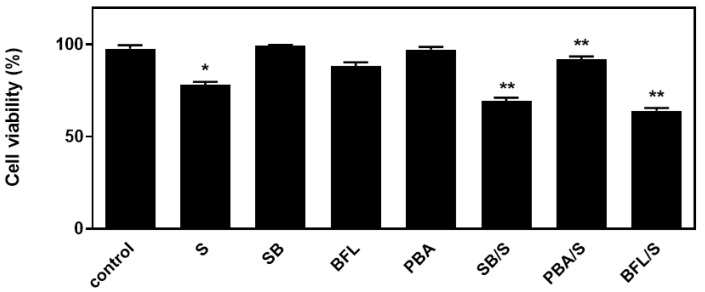
Assessment of H9c2 (%) cell viability by Trypan Blue exclusion assay. H9c2 cells were left untreated (control), treated with the inhibitor compounds alone (SB203580-SB, bafilomycin-BFL, PBA) or with the inhibitors followed by exposure to sorbitol (S). All experiments were performed in triplicate. Values are means ± SEM for at least three independent experiments. * *p* < 0.05 compared to control values; ** *p* < 0.01 compared to sorbitol-treated cells in the absence of the inhibitors.

In an attempt to probe into the role of the aforementioned signaling mechanisms activated by hyperosmotic stress in H9c2 cells, the effect of a number of compounds on cellular viability was next investigated. Accordingly, we used: SB203580 (SB-10 μM), the p38-MAPK inhibitor, bafilomycin (BFL-50 nM), inhibitor of late phase autophagy and 4-phenylbutyric acid (4-PBA-5 mM), inhibitor of ER stress [6]. Performing the trypan blue exclusion assay, we observed that exposure of H9c2 cells to 0.5 M sorbitol for 1 h considerably reduced their viability to 78.05 ± 0.98%. Of note, pre-incubation with SB203580 further reduced H9c2 viability (69.3 ± 0.91%), which was also the case when cells were pre-incubated with bafilomycin (64.0 ± 0.77%). Pre-incubation with PD98059 or SP600125 had no effect on H9c2 cells viability (data not shown). On the other hand, pre-incubation with 4-PBA conferred a salutary effect, significantly enhancing cellular viability (92.5 ± 0.5%) (Figure 9, *p* < 0.01 compared to sorbitol-treated cells in the absence of the inhibitors). Interestingly, exposure of H9c2 cells to 0.5 M sorbitol for a prolonged time interval led to a significant reduction in cellular viability that none of the aforementioned compounds were able to attenuate, demonstrated by the MTT analysis performed (data not shown).

**Figure 10:**
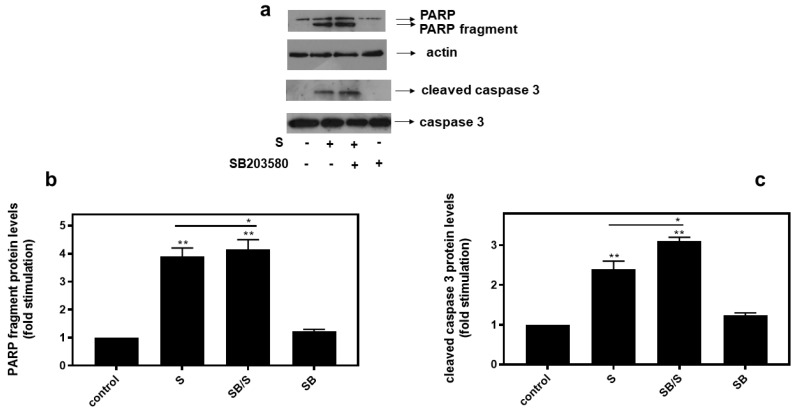
Inhibition of p38-MAPK, by SB203580, enhances hyperosmotic-stress-induced apoptotic markers in H9c2 cardiac cells. H9c2 cells were left untreated (control) or were incubated with SB203580 (SB) alone or with SB203580 followed by exposure to 0.5 M sorbitol in the presence of the inhibitor (SB/S). Cell extracts (40 μg/lane) were subjected to SDS-PAGE in 12% (*w*/*v*) polyacrylamide gels and immunoblotted with antibodies against endogenous levels of full-length PARP as well as the large 89 kDa PARP fragment ((**a**) upper panel) and of full length ((**a**) bottom panel) as well as cleaved caspase 3 ((**a**) 3rd panel from top). Equal protein loading was verified by immunoblotting identical samples with a specific anti-actin antibody ((**a**) 2nd panel from top). Western blots are representative of at least three independent experiments with overlapping results. Immunoreactive bands were quantified by laser scanning densitometry and plotted (**b**,**c**). Results are means ± SEM for at least three independent experiments. ** *p* < 0.01 compared to control values; * *p* < 0.05 compared to sorbitol-treated cells in the absence of SB203580.

**Figure 11:**
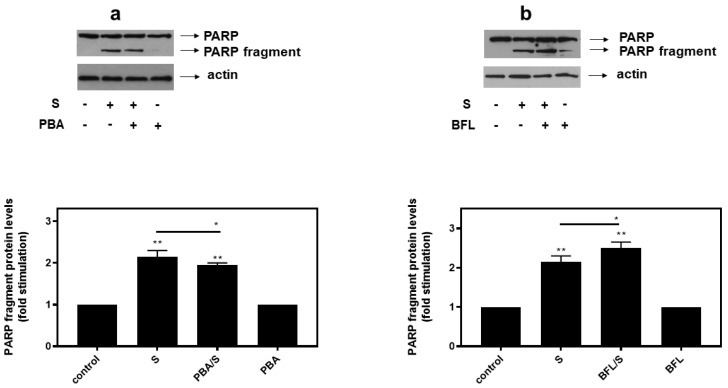
Opposite effects of PBA, an ER stress inhibitor and bafilomycin (BFL), an inhibitor of autophagy, on hyperosmotic-stress-induced PARP cleavage. H9c2 cells were left untreated (control) or were incubated with the inhibitors alone, or with the inhibitors followed by exposure to 0.5 M sorbitol in the presence of the inhibitors. Cell extracts (40 μg/lane) were subjected to SDS-PAGE in 8% (*w*/*v*) polyacrylamide gels and immunoblotted with antibodies against endogenous levels of full-length PARP as well as the large 89 kDa PARP fragment ((**a**,**b**) upper panels). Equal protein loading was verified by immunoblotting identical samples with a specific anti-actin antibody ((**a**,**b**) bottom panels). Western blots are representative of at least three independent experiments with overlapping results. Immunoreactive bands were quantified by laser scanning densitometry and plotted (respective graphs). Results are means ± SEM for at least three independent experiments. ** *p* < 0.01 compared to control values; * *p* < 0.05 compared to sorbitol-treated cells in the absence of the inhibitors.

In agreement with these findings, pre-treatment of H9c2 cells with SB203580 enhanced hyperosmotic-stress-induced apoptosis, that was previously observed to be triggered in our experimental setting under the conditions investigated [24], as evidenced by the increased levels of PARP fragmentation (Figure 10a, upper panel and b) and caspase 3 cleavage (Figure 10a, third panel and c). Equal protein loading was verified by immunodetection of actin (Figure 10a second panel). In addition, 4-PBA reduced PARP proteolysis in H9c2 cells exposed to 0.5M sorbitol (Figure 11a), while bafilomycin markedly augmented PARP fragmentation levels (Figure 11b).

### 3.5. Induction of p38-MAPK Phosphorylation and Autophagy by Hyperosmotic Stress Is ER-Stress-Independent in H9c2 Cells

**Figure 12:**
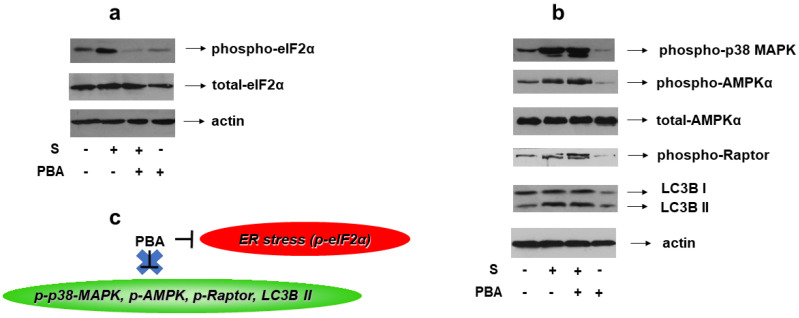
Effect of ER stress inhibition on hyperosmotic-stress-induced responses in H9c2 cells. H9c2 cells were left untreated or were incubated with PBA alone, or with PBA followed by exposure to 0.5 M sorbitol in the presence of PBA. Cell extracts (40 μg/lane) were subjected to SDS-PAGE and immunoblotted with antibodies against phosphorylated eIF2α ((**a**) upper panel), total levels of eIF2α ((**a**) second panel), phosphorylated p38-MAPK ((**b**) upper panel), phosphorylated AMPKα ((**b**) 2nd panel from top), total levels of AMPKα ((**b**) 3rd panel from top), phosphorylated Raptor ((**b**) 4th panel from top) and endogenous levels of total LC3B protein (LC3 I and LC3 II forms) ((**b**) 5th panel from top). Equal protein loading was verified by immunoblotting identical samples with a specific anti-actin antibody ((**a**,**b**) bottom panels). Western blots are representative of at least three independent experiments with overlapping results. (**c**) Schematic representation of the effect of ER stress inhibition by PBA under hyperosmotic stress conditions: PBA (an ER stress inhibitor evidenced by suppression of eIF2α phosphorylation) does not modify the phosphorylation status of p38-MAPK, AMPK or Raptor, nor the LC3B II / LC3B I ratio.

Given the reported association of ER stress with osmotic equilibrium disturbance [6], we set out to identify its potential involvement in the signaling pathways activated by 0.5 M sorbitol in H9c2 cells. To this end, 4-PBA, an established chemical chaperone and ER stress inhibitor, was used once again. We first verified its potential to abrogate hyperosmotic-stress-induced eIF2a phosphorylation (Figure 12a, upper panel). Subsequently, pretreatment of H9c2 cells with 4-PBA followed by exposure to 0.5 M sorbitol, was not found to modify the phosphorylation status of: p38-MAPK (Figure 12b, upper panel), AMPKα (Figure 12b, second panel) or Raptor (Figure 12b, fourth panel), nor the LC3B II /LC3B I ratio (Figure 12b, fifth panel). Equal protein loading was verified by immunoblotting with an anti-actin antibody (Figure 12b, bottom panel), while AMPKa total protein levels remained unchanged under these interventions (Figure 12b, third panel).

### 3.6. Suppressing Late-Phase Autophagy Does Not Affect Phosphorylation of p38-MAPK nor of eIF2α under Conditions of Hyperosmotic Stress in H9c2 Cells

**Figure 13:**
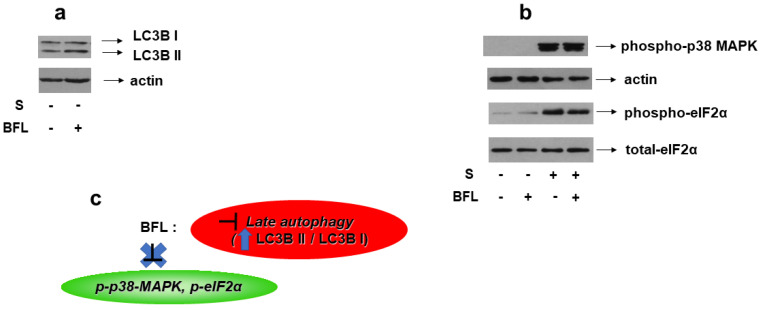
Effect of autophagy inhibition on hyperosmotic-stress-induced responses in H9c2 cells. H9c2 cells were left untreated or were incubated with bafilomycin (BFL) alone, or with BFL followed by exposure to 0.5 M sorbitol in the presence of BFL. Cell extracts (40 μg/lane) were subjected to SDS-PAGE and immunoblotted with antibodies against endogenous levels of total LC3B protein (LC3 I and LC3 II forms) ((**a**) upper panel), phosphorylated p38-MAPK ((**b**) upper panel), phosphorylated eIF2α ((**b**) 3rd panel from top), total levels of eIF2α ((**b**) bottom panel). Equal protein loading was verified by immunoblotting identical samples with a specific anti-actin antibody ((**a**) bottom panel and (**b**) 2nd panel from top). Western blots are representative of at least three independent experiments with overlapping results. (**c**): Schematic representation of the effect of late autophagy inhibition by bafilomycin under hyperosmotic stress conditions: BFL (which is a late autophagy inhibitor evidenced by upregulation of the LC3B II/LC3B I ratio) does not modify the phosphorylation status of p38-MAPK or eIF2α.

Bafilomycin A1 is a macrolide antibiotic that disrupts the fusion of autophagosome–lysosome, hence, functioning as an inhibitor of late-stage autophagy [25]. Consequently, bafilomycin promotes LC3B II formation, an effect that we also verified in our experimental setting (Figure 13a, upper panel). Of note, pretreatment of H9c2 cells with bafilomycin followed by exposure to 0.5 M sorbitol had no effect on p38-MAPK phosphorylation levels (Figure 13b, upper panel). Equal protein loading was verified by immunoblotting with an anti-actin antibody (Figure 13b, second panel). Interestingly, hyperosmotic-stress-induced eIF2α phosphorylation also remained unaffected in the presence of bafilomycin (Figure 13b, third panel). Immunoblotting analysis with an antibody against eIF2α total levels revealed their constitutive expression (Figure 13b, bottom panel).

## 4. Discussion

With osmotic stress associated with a number of pathological conditions, including metabolic disorders [26], bowel or liver diseases [27] and cardiovascular disorders [28], it is of immense significance to shed light on the osmoadaptive signaling mechanisms triggered to counteract the detrimental effects elicited. Having previously examined the role of aquaporins in the apoptosis triggered in H9c2 cardiac cells exposed to 0.5 M sorbitol [24], in the present study, we set out to investigate and identify key players and effectors of the compensatory responses stimulated.

Under hyperosmotic stress conditions, cells resort to suppression of the initiation step of mRNA translation [29]. This is partly accomplished via inactivation of translation initiation factor eIF2, via phosphorylation of its alpha subunit on serine 51 [30]. Accordingly, treatment of H9c2 cells with 0.5 M sorbitol was found to induce phosphorylation of eIF2α on Ser51 in a time-dependent manner (Figure 1a). *Of note, to our knowledge, this is the first report of hyperosmotic-stress-stimulated eIF2α phosphorylation in cardiac cells.* Interestingly, Bevilacqua et al. reported that while mild osmotic stress does not induce phosphorylation of eIF2α, the initiation factor is phosphorylated under severe osmotic stress conditions, also promoting apoptosis in mouse embryonic fibroblasts (MEFs) and primary cortical neurons [29]. In correlation with our findings, Bevilacqua et al. also noted a prolonged eIF2α phosphorylation pattern in MEFs that was not, however, triggered as rapidly as in our setting of H9c2 cells [29]. This difference could indicate that cardiac cells are more sensitive in sensing and responding to severe osmotic perturbations, thereby directly blocking translation to restore homeostasis and preserve heart function.

Phosphorylation of eIF2α constitutes a fundamental step in the Integrated Stress Response (IRS), as well as in the Unfolded Protein Response (UPR) [31]. Both IRS and UPR constitute adaptive mechanisms activated by diverse stress stimuli, including endoplasmic reticulum (ER) stress [32]. Thus, we subsequently investigated whether treatment of H9c2 cells with 0.5 M sorbitol induces activation of ER stress. Accordingly, we detected a considerable and long-lasting augmentation in the expression levels of Glucose-Regulated Protein 78 (GRP78), otherwise termed as BiP, illustrated in Figure 1b. Levels of BiP are increased under conditions of ER stress to counterbalance the deleterious effects of the latter, through restoration of protein folding and assembly [33]. Our findings are in agreement with studies underlining ER stress stimulation by hyperosmotic conditions in rat hypothalamus [34], SK-N-SH human neuroblastoma cells and in obese rats’ retina [35], as well as in adult rat cardiac myocytes [6].

Accumulating data highlight the interrelation between ER stress and autophagy [36]. In particular, Petrovski et al. showed that depending on the nature, intensity and duration of the ER stress, the autophagy induced can determine cell fate by exerting either beneficial or detrimental effects in the context of the ischemic heart [37]. In addition, Wang et al. also marked induction of autophagy under ER stress conditions, in rat primary neurons and pheochromocytoma (PC12) cells, evidenced by LC3-I to LC3-II conversion and upregulation of AMPK phosphorylation levels [36]. In addition, Pena-Oyarzun et al. noted the stimulation of autophagy in human HeLa and HCT116 cell lines under hyperosmotic conditions [1]. Corroborating these studies, as presented in Figure 2, treatment of H9c2 cells with 0.5 M sorbitol also enacted the autophagic mechanism, evidenced by the increase in: (i) LC3-II/LC3-I ratio and (ii) Beclin-1 protein expression levels.

Beclin-1 has been shown to increase in parallel with LC3 II, preserving cardiac mitochondrial integrity during sepsis [8]. Further supporting our findings, Guan et al. noted that both ER stress and autophagy mediate H9c2 response to hypoxia/reoxygenation injury [38]. What is more, Chen et al. also marked the cardioprotective role of autophagy against ER stress in neonatal cardiac myocytes [9]. In accordance with the notion that the mechanisms regulating promotion of autophagy may also involve activation of the AMPK pathway [39], we detected phosphorylation of AMPK at Thr172, indicating its activation [40] (Figure 3a). This profile matched the pattern of Raptor phosphorylation (at Ser792), which is known to result in mTORC1 suppression, hence, further promoting autophagy [41] (Figure 3b). In line with our study, hyperosmotic stress amplified AMPK phosphorylation in human colon tumor cell line HCT116 and human cervical cancer cell line HeLa [1]. *It should be pointed out that, to our knowledge, this is the first report of AMPK and Raptor time-dependent phosphorylation profiles, under hyperosmotic stress conditions, in cardiac cells.*

With eIF2α characterized as a “master regulator of the stress response” [29], we next set out to gain insight into the particular effectors regulating its phosphorylation by 0.5 M sorbitol in H9c2 cells. Given that MAPKs play a predominant role in osmotic stress signal transduction mechanisms [21], we first confirmed phosphorylation, thus, activation, of all the three best-characterized members of this kinase superfamily in our experimental setting (Figure 4 and Figure 5). We subsequently examined their involvement in eIF2α phosphorylation, using selective pharmacological MAPKs inhibitors. As shown in Figure 6, no interplay between p38-MAPK, ERK1/2 or JNK1/2 and phospho-eIF2α was identified. This finding contradicts reports demonstrating p38-MAPK to mediate eIF2α phosphorylation in granulosa cells and oocyte complexes [42], as well as in podocytes [43] and human gastric adenocarcinoma cell lines [44] under conditions of ER stress. Our data are also in disagreement with a study by Zhong et al., manifesting ERK1/2 as well as JNK1/2 involvement in eIF2α phosphorylation in UV-irradiated mouse epidermal jb6 cells [45].

An effort was next made to assess the potential causative relationship between eIF2α phosphorylation and elevated levels of ROS. The deleterious effect of hyperosmotic stress conditions has been linked to excessive generation of ROS [46,47], which have also been implicated in ER stress induction [48].

In this context, we initially confirmed increased ROS generation in H9c2 cells treated with 0.5 M sorbitol, as evidenced by enhanced DCFH-DA fluorescence (Figure 7). Oxyblot analysis also revealed augmented levels of carbonyl groups in protein side chains under the conditions investigated (Figure 7). Keeping in mind that potential pitfalls and advantages exist for most of the methods routinely applied to establish ROS generation in cells and tissues, one should consider these limitations when interpreting the respective data. Accordingly, while DCFH-DA is one of the most widely used probes in studies of redox-associated signaling mechanisms, the complex intracellular redox chemistry of this compound may lead to misleading interpretations, particularly as far as quantitative evaluation of hydrogen peroxide is concerned. Thus, in future studies, our data could be further substantiated, by using recently developed fluorogenic probes that could also reveal the specific source and subcellular compartment responsible for the enhanced hyperosmotic-stress-induced ROS levels observed.

Interestingly, apocynin (NOX inhibitor) and HOE642 (selective NHE-1 inhibitor) both abrogated eIF2α phosphorylation under hyperosmotic stress conditions (Figure 8). This observation is in agreement with reports identifying NOX as a primary source of ROS in the cardiovascular system and underlining regulation of NHE-1 activity by osmotic perturbations [49]. This is also in accordance with a study by Guo et al., marking activation of ER stress downstream of oxidative stress, leading to myocardial dysfunction [50]. What is more, Dalal et al. reported NOX involvement in the oxidative stress triggered under ER stress conditions, in adult mouse ventricular myocytes [51]. *To our knowledge, the mediatory role of NOX as well as of NHE1 on eIF2α phosphorylation, indicated in our study, has not been previously reported. The pathophysiological implications of this novel association deserve further investigation, since NOX and NHE-1 are known for their involvement in vascular contraction, cardiovascular disorders, arrythmias, myocardial stunning, hypertension, etc. [49].*

With the preservation of myocardial function largely dependent on cardiac myocytes viability under stress conditions, we subsequently evaluated the effect of various compounds on the H9c2 cells’ viability after treatment with 0.5 M sorbitol. Strikingly, the detrimental effect of treating H9c2 cells with 0.5 M sorbitol for 1 h was further accelerated in the presence of SB203580 (a p38-MAPK inhibitor) and bafilomycin (an inhibitor of autophagy), while 4-PBA (an ER stress inhibitor) partially alleviated it (Figure 9). The data illustrated in Figure 10 and Figure 11 further support the aforementioned findings.

These results indicate a salutary role for p38-MAPK and autophagy under the hyperosmotic conditions investigated. However, “at the end of the day” this beneficial input elicited by activation of p38-MAPK and autophagy pathways has been proven insufficient to counterbalance the overwhelming deleterious effect of exposure of H9c2 cells to 0.5 M sorbitol over time [3,24]. Contradictory reports on the role of p38-MAPK range from conferring cardioprotection to favoring apoptosis and differ by circumstance [52]. Thus, identifying signaling molecules involved in p38-MAPK regulation may provide new targets for therapeutic pharmacological interventions. Therefore, further studies applying other state-of-the-art experimental approaches could contribute to the identification of the effectors involved in the p38-MAPK signaling pathway, triggered by hyperosmotic stress in H9c2 cells. This could pave the way to analysis of the activation mechanism initiated, to determination of the specific p38-MAPK isoforms activated, as well as to assessment of the substrates these kinases interact with, ultimately specifying their functional role.

On the other hand, disruption of autophagy by bafilomycin has been demonstrated to have an equally aggravating effect. In particular, bafilomycin has been found to ablate the protection elicited by mitochondrial aldehyde dehydrogenase 2 (ALDH2) under hyperglycemic conditions and to cancel off the protection conferred by eicosapentaenoic acid against lipotoxicity in H9c2 cells ([53,54], respectively). Other inhibitors of autophagy have also been found to enhance the cell death conferred by ER stress [55]. In line with our observations, indicating ER stress involvement in 0.5 M sorbitol-induced cell death, 4-PBA has been shown to suppress hyperglycemia (25 mM glucose)-induced cell death in atrial cardiomyocytes [56], as well as hyperosmotic medium (131 mM mannitol)-stimulated cell death in adult rat cardiac myocytes [6].

Evidently, cardiac cells resort to different strategies to preserve myocardial function under stress conditions [57]. Investigating the potential interplay and functional links between the signal transduction mechanisms activated is of extreme importance since it may reveal protein interactomes and networks, as well as novel biological roles of the effectors involved. Consequently, given the notion that ER stress, autophagy, apoptosis and p38-MAPK have been occasionally reported to interrelate and crosstalk [9,43,44], an effort was next made to decipher their potential interaction in our H9c2 model.

As illustrated in Figure 12, inhibition of ER stress by 4-PBA did not hinder hyperosmotic-stress-induced phosphorylation of p38-MAPK, AMPKα and Raptor, nor LC3B I to LC3B II conversion. Therefore, in our experimental model, the ER stress stimulated by 0.5 M sorbitol is not involved in p38-MAPK activation nor in the induction of autophagy.

In agreement with our observation, Cardoso et al. [43] demonstrated that although ER stress and p38-MAPK are both activated by angiotensin II in mouse podocytes, there is no direct association between them. On the other hand, Li et al. observed ER stress to trigger p38-MAPK activation in the mouse brain, in an animal model of Parkinson disease, as well as a form of autophagy termed chaperone-mediated autophagy [58]. Furthermore, in contrast to our findings, Gao et al. found 4-PBA to inhibit upregulation of autophagy markers in SH-SY5Y cells exposed to H_2_O_2_ [59]. Nevertheless, in MEF cells, although induction of ER stress was demonstrated to enhance autophagy, no involvement of AMPKα was demonstrated [60]. These discrepancies could be attributed to the cell types involved, as well as to the nature of the stimulus disturbing cellular homeostasis.

Given that the contribution of autophagy to p38-MAPK activation and initiation of the ISR under conditions of hyperosmotic stress remain unresolved, the effect of bafilomycin on these effectors was next investigated. Suppression of autophagy in the presence of bafilomycin was verified by the upregulation in the LC3-II/LC3-I ratio (Figure 13a, upper panel). Interestingly, bafilomycin did not have a discernible effect on 0.5 M sorbitol-induced phosphorylation of p38-MAPK nor of eIF2α (Figure 13b, upper and third panels, respectively). To our knowledge, *this is the first time that the causative relation between induction of autophagy and p38-MAPK activation has been investigated in cardiac cells*, since most studies probe into the reverse, revealing the inhibitory effect of p38-MAPK suppression on upregulated autophagic markers [61,62]. Contradicting our results, Yu and Long reported that inhibition of autophagy by bafilomycin triggers phosphorylation of eIF2α in C2C12 myotubes [25]. Nevertheless, the majority of reports examine the predominant role of ER stress in triggering autophagy rather than the reverse [37].

**Figure 14:**
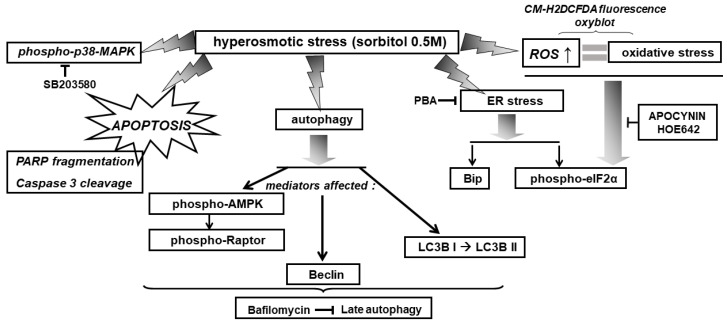
Schematic diagram of a model illustrating the diverse signaling mechanisms triggered by hyperosmotic stress in H9c2 cells, with emphasis on the effectors activated, mediating the observed responses. → activation, ˧ inhibition.

**Figure 15:**
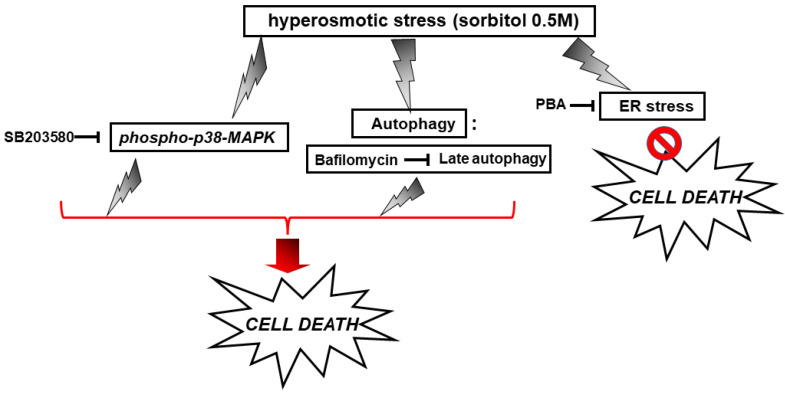
Scheme depicting the effect of SB203580, bafilomycin and PBA on H9c2 cell viability under conditions of hyperosmotic stress. 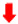 enhancement, 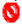 suppression.

Overall, exposure of H9c2 cells to 0.5 M sorbitol was found to induce: the ISR, ER stress, autophagy, as well as p38-MAPK activation, in a complementary but independent and nonreciprocal manner (depicted in Figure 14 and Figure 15).

## 5. Conclusions

In the present study, hyperosmotic stress (0.5 M sorbitol) has been found to exert detrimental effects on H9c2 cell viability, triggering multiple signaling pathways. In particular, our findings revealed the NOX- and NHE-1-mediated time-dependent phosphorylation of eIF2α, the time-dependent phosphorylation of AMPK and Raptor, along with ROS generation. Interestingly, while ERS was established to mediate the deleterious effect of sorbitol, p38-MAPK and late phase autophagy partially “counteracted” its overwhelming effects. 

Deciphering the network of signal transduction mechanisms triggered under stress conditions that jeopardize proper myocardial function is of fundamental importance. This kind of studies can bear significant implications on the design of appropriate prevention schemes, identification of diagnostic and prognostic targets, re-evaluation of therapeutic strategies implemented, ultimately affecting clinical practice regarding cardiovascular diseases.

## Data Availability

The data presented in this study are available on request from the corresponding author.

## Abbreviations

BSA	bovine serum albumin
DMSO	dimethyl sulphoxide
DTT	ditheiothreitol
ECL	enhanced chemiluminescence
ERS	endoplasmic reticulum stress
ISR	Integrated stress response
MAPK	mitogen-activated protein kinase
PARP	poly(ADP-ribose) polymerase
ROS	reactive oxygen species
UPR	unfolded Protein Response
TBS	tris-buffered saline
